# Positive Affect Predicts Turnover Intention Mediated by Online Work Engagement: A Perspective of R&D Professionals in the Information and Communication Technology Industry

**DOI:** 10.3389/fpsyg.2021.764953

**Published:** 2021-12-20

**Authors:** Jon-Chao Hong, Sirirat Petsangsri, Yuting Cui

**Affiliations:** ^1^Institute for Research Excellence in Learning Science, National Taiwan Normal University, Taipei City, Taiwan; ^2^Department of Industrial Education, King Mongkut’s Institute of Technology Ladkrabang, Bangkok, Thailand; ^3^Institute of Vocational and Adult Education, Faculty of Education, Beijing Normal University, Beijing, China

**Keywords:** positive affect, work engagement, turnover intention, organizational behavior, remote working

## Abstract

Remote work has become the most popular approach during the COVID-19 lockdown; however, remote work engagement is an issue which creates challenges for human resource management. Some individuals engage in work no matter how difficult the job is, but some people’s minds wander, no matter how simple the job is. To address this issue, this study drew on trait activation theory, which indicates that one’s positive disposition may affect one’s turnover intention mediated by work engagement, to formulate a research model to test the associations among R&D professionals. Questionnaires were distributed to R&D professionals working in China information and communication technology (ICT) through several Instant Message groups. In total, 386 valid questionnaires were collected for confirmatory factor analysis with structural equation modeling to verify the research model. The study found that positive affect can positively predict three types of remote work engagement: the cognitive, emotional, and behavioral engagement of R&D personnel. All three types of remote work engagement of R&D personnel can negatively predict their turnover intention. The results suggest that if human resource managers working in the ICT industry want to reduce the turnover intention rate of R&D workers under pressure from COVID-19, they should enhance workers’ remote engagement by selecting R&D workers with a high level of positive affect.

## Introduction

The SARS-COV-2, COVID-19, is becoming more virulent (Huang et al., 2020), and the associated pandemic is creating huge challenges for those working from home, affecting employees’ psychological pressure in a way that has never before been seen (Carnevale and Hatak, 2020). It has created a situation of crisis and uncertainty, during which companies have to provide adequate information and communication technology (ICT) to foster higher levels of engagement in online work with measures to prevent employee stress (Ruiz-Frutos et al., 2021). It is possible that the COVID-19 pandemic will result in a number of work paradigm shifts within organizations which are facing new demands, leading to a “new normal” of working from home (Howe et al., 2020). With the aim of gaining insights from a case of online work, the focus of this study was to understand the potential factors that influence online work for R&D professionals in ICT jobs during the COVID-19 lockdown.

The high costs of turnover are associated with recruitment and the difficulties of finding talented employees (International Labour Organization, 2020). For example, one study found that the cost of replacement was on average 213% of the annual salary of highly skilled employees (Purvis, 2021). R&D professionals as highly skilled workers in the ICT industry face intense pressure to innovate and to create groundbreaking products (Jiang et al., 2019). Their turnover has been identified as a major challenge for companies that wish to retain workers. Examples of measures to retain such workers include creating an attractive work culture by providing flexible work schedules (Caillier, 2018) and mentoring programs (Fogarty et al., 2017). According to the job demand-resource theory, every occupation has two general categories of job demands and resources which may improve employee well-being and performance (Bakker and Demerouti, 2007) and reduce turnover intention (Islam et al., 2018). While turnover intention cannot replace actual turnover, it is correlated and can serve equally well as a precursor to actual leaving (Dalton et al., 1999; Lee H.-F. et al., 2019). Considering the change of job demand-resource associated with turnover intention cost during the COVID-19 pandemic, this study aimed to understand the individual turnover intention of ICT personnel.

Trait activation theory (TAT; Tett et al., 2013) states that work engagement is the activated state of positive work-related affect with increased job motivation (e.g., Warr and Inceoglu, 2012). Generally, high job motivation means having a positive mental state while working, and having high job motivation while engaging in a job is a particularly important variable in performance (Jung et al., 2021). Considering this, the present study took positive affect as an individual factor related to experiences in R&D jobs. Moreover, previous studies have focused on employees’ turnover intention as an outcome of their work engagement (Jiang et al., 2019), but few studies have as yet examined how turnover intention is influenced by positive affect, mediated by online work engagement. Thus, this study aimed to fill this gap by developing a research model that explores the mediating role of work engagement in relation to R&D professionals’ positive affect and turnover intention.

## Literature Review

### Turnover Intention

DeTienne et al. (2012) stated that, “Turnover intention is simply whether an employee has the objective of self-terminating his or her employment” (p. 380). Recruitment, selection, and training of human resource management (HRM) that represent a heavy investment for a firm. Previous meta-analyses aggregated that the estimates of the correlations between turnover intention and actual turnover ranged from 0.31 to 0.52 (Dalton et al., 1999). Cho and Lewis (2012) used the central personnel data file (CPDF) and MPS 2005 to assess how well turnover intention predicts turnover in the federal sector. Their study indicated that turnover intention seemed to be a reasonable proxy for actual turnover, especially as using age and experience as units of analysis resulted in a correlation of 0.7 or higher. Thus, turnover intentions have a direct effect on actual turnover (Cohen et al., 2016), although the estimate coefficient may be limited for different groups.

During the COVID-19 pandemic, working environments have faced uncertainty as a result of technological changes, economic fluctuations, and political insecurity. It has therefore been impossible for organizations to guarantee employment stability for all of their employees (Bajrami et al., 2021). Considering that job insecurity is a risk factor, its effects on turnover intention are a managerial factor during the COVID-19 pandemic (Jung et al., 2021). The job demand and control models suggest that ICT employees’ ability to pace themselves and to make their own task decisions can reduce their turnover intention (Shih et al., 2011). Particularly, R&D jobs are usually designed to give the ICT employees a great deal of autonomy in terms of the pace and process of their work. However, R&D workers in the ICT industry need to work smart and hard, and their turnover intention can impact the competitiveness of ICT firms. Yet, during the crisis of the COVID-19 pandemic, R&D workers’ turnover intention has not yet been clearly studied. It was therefore explored in this study.

### Work Engagement

Work engagement (WE) has been defined in two main ways in the literature. Kahn (1990) originally defined it as “the harnessing of organization members’ selves to their work roles; in engagement, people employ and express themselves physically, cognitively, and emotionally during role performances” (p. 694). These three types of WE were therefore adopted in this study. The advances in applied ICT have led to increased flexibility in terms of how and where people can work. Thus, this study considered R&D workers in the context of the electronics industry to explore their three types of WE based on Kahn’s (1990) classification.

It has been argued that if workers perceive their jobs as being both significant and meaningful, and if they experience a sense of pride while performing work-related activities, their three types of WE will be promoted (Perera et al., 2018). Recent studies related to WE have been thoroughly discussed in terms of the environments or organizational factors, such as in the hospitality industry (Dai et al., 2021), and in the medical industry (Cao et al., 2019). However, few studies have considered WE in the ICT industry to explicate the motivation of R&D professionals. Additionally, with the spread of COVID-19, working remotely quickly became the norm for workers in numerous organizations (Shafi et al., 2020), with employees having to adjust to teleworking from home (Burdorf et al., 2020). If this remote work was not initially employed, the ability to work effectively in a virtual format may have created a hurdle for employees (Howe et al., 2020). The present study therefore aimed to understand more about the WE of R&D professionals while working from home.

### Positive Affect in the Context of the Workplace

The term “affect” tends to be used as an umbrella term for mood trait, or in other words, it refers to affection that is not directed toward a specific object, for example, feeling tired versus feeling alert (Reis et al., 2016). Positive affect is related to a subjective experience of emotions, moods, or dispositions which have a facilitating effect on behaviors and interactions with environments (Jones and Graham-Engeland, 2021). Here, we use the term affect to refer to the psychological trait that shapes the states of one’s feelings in the face of failure.

As the COVID-19 pandemic and interventions continue to have an impact on everyone’s daily life, personality research may be useful for addressing the various ways in which different people respond to a major global health crisis such as this (Hardin et al., 2021). By investigating the ways in which psychological traits predict how people respond to unprecedented shifts in their environment, research on the role of personal traits in the pandemic may be able to make a broader contribution to personality psychology (Zajenkowski et al., 2020). Thus, we draw upon the task-specific affective responses as a personal trait which is generated in the process of R&D workers interacting with their remote job activities while facing the pandemic crisis.

## Hypotheses

### Positive Affect and Remote Work Engagement

The openness of positive emotion may allow individuals to attach a more positive valence to neutral experiences, thus enhancing the positive affect even further (Huffziger et al., 2013). In other words, promoting greater positive affectivity may broaden one’s outlook and build psychological resources that can promote healthy emotional responses in the workplace (Fredrickson, 2004). Under emotion-eliciting processes, positive affect can validate the prediction of motivation to WE (Laguna, 2019; Yan et al., 2019). A workplace culture which involves continually being “on,” and which leaves no opportunity to experience the pleasure of thinking cognitively and deeply can affect job engagement (Collins, 2017). Specifically, personality has been linked to health-risk perceptions, whereas some personality traits have positively predicted job behaviors (Malesza and Kaczmarek, 2019). Accordingly, during the COVID-19 pandemic, how positive affect as a personal trait is related to remote WE was hypothesized as follows:

H1: Positive affect is positively related to cognitive engagement in the context of working remotely;

H2: Positive affect is positively related to emotional engagement in the context of working remotely;

H3: Positive affect is positively related to behavioral engagement in the context of working remotely.

### Remote Work Engagement and Turnover Intention

It has been shown that there is a relationship between individual differences in job engagement and outcomes such as organizational commitment or weaker turnover intentions (Meyers et al., 2020). Turnover intention is recognized as an important factor as it relates to WE (Fu and Chen, 2015). For instance, previous studies found that the affective dimension of WE has a strong link to the turnover intention of Malaysian nurses (Al-Hussami et al., 2014). Moreover, Shin and Jeung (2019) found that allowing employees to actively participate in their work can effectively reduce their willingness to leave their job. Therefore, to avoid excessive turnover intentions, employers should pay attention to employees’ behavioral engagement. While working from home during the COVID-19 pandemic, how remote WE is related to the turnover intentions of R&D personnel in the ICT industry is hypothesized as follows:

H4: In the context of working remotely, emotional engagement is positively related to turnover intention;

H5: In the context of working remotely, cognitive engagement is positively related to turnover intention;

H6: In the context of working remotely, behavioral engagement is positively related to turnover intention.

### Positive Affect and Turnover Intention

Previous studies have found that monitoring employees with high levels of positive affect can encourage them to remain in the company (Steel and Lounsbury, 2009). Studies have already demonstrated that intentions to leave can be changed by a mediation process (e.g., Afzal et al., 2019; Cepale et al., 2021). Several studies revealed that there may be a mediating process, respectively, with Afzal et al. (2019) and Cepale et al. (2021) supporting the existence of mediating variables (e.g., self-efficacy, organizational socialization, and organizational identification) between affect and intention to leave through cross-sectional study and three-waves (or longitudinal) models. WE can mediate the relationship between a high level of positive feeling and organizational commitment (Teo et al., 2020). Moreover, Gottschalck et al. (2020) highlighted that if organizations can consider individual differences in the patterns of turnover intentions, they can design retention strategies that are more closely aligned with the specific needs of different employee groups. Accordingly, how R&D professionals’ positive affect is related to their turnover intention mediated by remote WE during COVID-19 was hypothesized as follows:

H7: Positive affect is negatively related to turnover intention mediated by remote WE.

### Research Model

**FIGURE 1:**
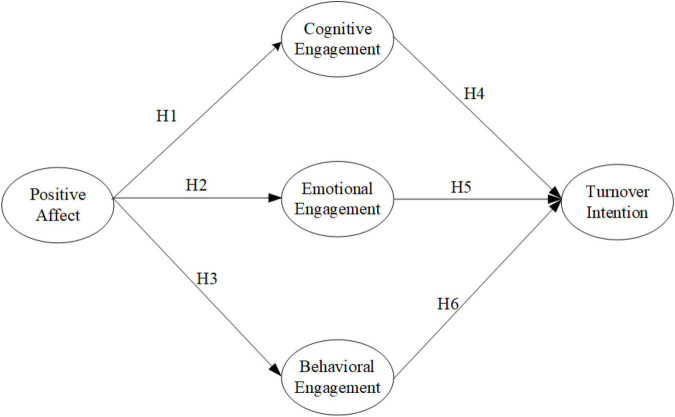
The hypothesized research model.

The appraisal theories of emotion (Scherer et al., 2001) have been used in numerous studies to evaluate the processes of emotion elicitation. Since these theories rely on cognitive processes such as perception, decision making, and behavior, they can be used to assess one’s feelings about a job. Ecological theories focus on the interaction of persons and environments requiring individual unique physical and psychological adaptations (Bronfenbrenner, 1995). This suggests that engagement plays an important role in generating behavioral responses (Jain and Asawa, 2019; McLaughlin et al., 2019); thus, during the COVID-19 lockdown, the research model for investigating the relationship between positive affect, remote WE, and turnover intention of R&D professionals was proposed as shown in Figure 1.

## Materials and Methods

### Participants and Procedure

Snowball sampling was adopted to recruit participants from among R&D professionals in information technology who had worked remotely for at least 3 months during the COVID-19 lockdown in China. Questionnaires were distributed by post on a number of Instant Messaging groups. Group members were asked to reply and to pass on the questionnaires to their colleagues on June 01, 2020. After 2 weeks, there were 412 returned questionnaires, of which 26 invalid questionnaires were excluded (19 were incompletely filled out, and 7 subjects had the same answer for all questions), so that 386 useful data were retained for analysis. Invalid data were randomly distributed across groups, meaning that they did not cause random bias in the model.

In terms of gender, 75.9% (293) were males and 24.1% (93) were females. Regarding age, participants up to 29 years old comprised 6.7% (26) of the sample, between 30 and 34 comprised 31.6% (122), between 35 and 39 comprised 25.9% (100), and 40 years old and above comprised 15.1% (58). As for the participants’ educational backgrounds, 14.7% (57) had a qualification of junior college or below, 45.1% (174) had a bachelor’s degree, and 40.2% (155) had a master’s degree or above. In terms of years of employment in their current R&D jobs, 6 years or less comprised 12.9% (50), between 7 and 9 years comprised 18.9% (73), between 10 and 12 years comprised 16.6% (64), between 13 and 15 years comprised 10.9% (42), between 16 and 18 years comprised 16.3% (63), and 19 years and above comprised 24.3% (94).

### Questionnaire

The content of the questionnaire was designed with reference to previous research and related theories; moreover, to ensure face validity (Hardesty and Bearden, 2004), the questionnaire content was reviewed by domain experts. After the data collection, the items and the face validity of the questionnaire were analyzed. Items were rated on a 5-point Likert scale (ranging from 1 = *very slightly or not at all* to 5 = *extremely*). This study adopted a confirmatory study approach, meaning that the reliability and validity of the constructs of the questionnaire should be re-examined, as suggested by Hair et al. (2019).

#### Positive Affect Measurement

PANAS (positive and negative affect scale; Watson et al., 1988) is used to assess broad-based positive and negative mood states (Watson, 2000). Joshanloo (2017) shortened the scale to 12 items for measuring positive and negative affect. Eight items were designed in this study to assess positive affect (e.g., “You don’t feel sad when your boss asks you to redo the work during the COVID-19 lockdown” and “You will be thankful when someone gives you some advice to improve your job during the COVID-19 lockdown.”).

#### Remote Work Engagement Measurement

The WE scale was developed by Walden et al. (2017) (Work Engagement Scale). Albro and McElfresh (2021) applied it to assess how librarians in a remote work environment coped with COVID-19. Adapted from their studies, to measure participants’ perceptions of the three types of engagement during the COVID-19 lockdown, six items for each WE were designed in this study. For example, for cognitive engagement: “When working remotely, I pay a lot of attention to my job” and “When working remotely, I devote a lot of attention to my job.” For emotional engagement: “When working remotely, I feel energetic when doing my job” and “When working remotely, I am enthusiastic about my job.” For behavioral engagement: “When working remotely, I exert my full effort to perform my job” and “When working remotely, I exert a lot of energy when performing my job.”

#### Turnover Intention Measurement

DeTienne et al. (2012) stated that, “turnover intention is simply whether an employee has the objective of self-terminating his or her employment” (p. 380). Adapted from the Chinese version of the Turnover Intention Scale developed by Su (2021), this study designed seven items to measure turnover intention (e.g., “During the COVID-19 lockdown, I will leave my job if another job becomes available” and “During the COVID-19 lockdown, I will probably not stay with this organization for much longer”).

## Results

### Confirmatory Factor Analysis for Measurement Model

The internal and external validity of each item was tested in this study. To perform the test of internal validity, first, the factor loading less than 0.5 was deleted. Second, first-order confirmatory factor analysis (CFA) was performed to test those values of χ*^2^/df*, RMSEA, GFI and AGFI, by deleting those items with highest residual values. If those values are reach the threshold suggested, then those items would be remained (Hair et al., 2019). Table 1 shows the results for internal validity, therefore, we reduced the number of items from eight to five for positive affect, from six to four for cognitive engagement, from six to four for emotional engagement, from six to five for behavioral engagement, and from seven to five for turnover intention.

**TABLE 1:** Results of first-order confirmatory factor analysis – model fit measures.

Index	Threshold	Positive affect	Cognitive engagement	Emotional engagement	Behavioral engagement	Turnover intention
χ*^2^/df*	<5	2.82	1.02	3.30	1.82	1.12
RMSEA	<0.10	0.07	0.09	0.08	0.05	0.08
GFI	>0.8	0.96	0.98	0.96	0.97	0.99
AGFI	>0.8	0.94	0.97	0.98	0.95	0.98
FL	>0.5	0.72–0.77	0.64–0.81	0.65–0.84	0.67–0.83	0.63–0.82
*t*-value	>10	13.59–18.75	17.43–20.71	15.55–20.43	17.61–23.16	10.66–15.99

To test the external validity of each item, the top 27% of the scale scores were classified as the high group, and the bottom 27% were selected as the low group for independent sample *t-*tests. A *t*-value above 10 (*p* < 0.001) is considered to be statistically significant (Awang et al., 2015). The *t*-value in this study was higher than 13.59 (*p* < 0.001), indicating that all questions in this study were discriminatory, that is, all items have external validity for use in different situations with different samples (Green and Salkind, 2004).

### Reliability and Validity Analyses

Awang et al. (2015) indicated that when the Cronbach’s α value and composite reliability (CR) fall between 0.70 and 0.98, there is a high level of reliability. Table 2 shows that the Cronbach’s α values are over 0.83, indicating that those constructs have good internal consistency; the CR values are over 0.70, revealing that those constructs have acceptable external consistency. To test the convergent validity of each construct, the values of AVE and the factor loading of each construct should be above 0.5 (Hair et al., 2019). Table 2 shows that the values of FL are over 0.68, and the values of AVE are over 0.63, indicating that all constructs have good convergent validity.

**TABLE 2:** Construct reliability and validity analysis (*n* = 386).

Constructs	*M*	*SD*	α	CR	FL	AVE
Positive affect	3.77	0.72	0.83	0.84	0.73	0.63
Cognitive engagement	3.75	0.76	0.85	0.83	0.72	0.70
Emotional engagement	3.77	0.71	0.84	0.84	0.68	0.67
Behavioral engagement	3.79	0.73	0.88	0.88	0.76	0.69
Turnover intention	2.54	0.70	0.84	0.87	0.75	0.68

To test the construct discriminant validity (i.e., the difference between two different constructs), Awang et al. (2015) suggested that the correlation coefficient between two constructs should be lower than the square root of the AVE of the construct. Table 3 shows that the ranges of the square root of the AVE of each construct are higher than the value of correlation coefficients between constructs, indicating that the questionnaire has good construct discriminative validity.

**TABLE 3:** Construct discriminative validity analysis (*n* = 386).

Constructs	1	2	3	4	5
(1) Positive affect	**(0.74)**				
(2) Cognitive engagement	0.66***	**(0.77)**			
(3) Emotional engagement	0.58***	0.72***	**(0.75)**		
(4) Behavioral engagement	0.60***	0.71***	0.74***	**(0.77)**	
(5) Turnover intention	−0.57***	−0.73***	−0.72***	−0.71***	**(0.75)**

***p < 0.001. Bold values on the diagonal are the square roots of AVE. To establish the discriminative validity, the value should be greater than the inter-construct correlations.

### Model Fit Analysis

Model fit is analyzed by AMOS 20.0, and is described in three parts. Hair et al. (2019) suggested that absolute fit measures, incremental fit measures, and parsimonious fit measures should be taken into consideration before path analysis.

Absolute fit measures: Hair et al. (2019) suggested that if *x^2^/df* = 2.61 is less than 5, RMSEA is less than 0.08, and GFI and AGFI are above 0.8, it is not a good model fit. In this study, the value of RMSEA is 0.07, GFI is 0.87, and AGFI is 0.84, indicating that all of the absolute fit measure values exceed those thresholds. Incremental fit measures: Hair et al. (2019) suggested that the values of NFI, TLI, CFI, IFI, and RFI should be larger than 0.8 to show that the model has a fair fit. In this study, NFI = 0.879, TLI = 0.91, CFI = 0.92, IFI = 0.92, and RFI = 0.87, so all of the incremental data are over the threshold, indicating a fair model fit. Parsimonious fit measures: The value of PNFI and PGFI larger than 0.5 means it has a good model fit (Hu and Bentler, 1999). In this study, PNFI = 0.79 and PGFI = 0.72, which were larger than the threshold 0.50, indicating that the Parsimonious fit measures have a good model fit. According to Podsakoff et al. (2003), Harman’s single-factor test is one of the most widely adopted techniques for determining the model fit of common methodological bias. The first factor interpreted 17.95% of the total variance which was below the threshold of 40%. This result indicates that there was no common method bias in this study.

### Path Analysis

**FIGURE 2:**
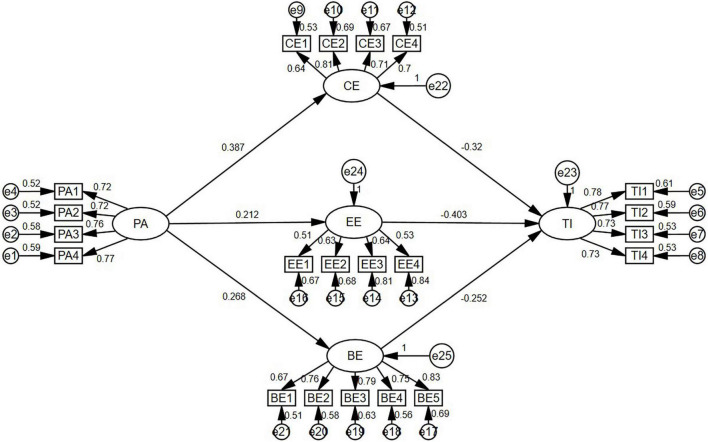
Model fit analysis.

In the verification step, this study adopted AMOS 20.0 for path modeling over the covariance-based SEM. Hair et al. (2012) suggested that the significance of a pathway is identified by each route coefficient’s value. Figure 2 shows that all of the hypotheses were supported as follows: positive affect was positively correlated to cognitive engagement (β = 0.387, *t* = 6.267, *p* < 0.001); positive affect was positively correlated to emotional engagement (β = 0.212, *t* = 3.707, *p* < 0.001); positive affect was positively correlated to behavioral engagement (β = 0.268, *t* = 4.543, *p* < 0.001); cognitive engagement was negativity correlated to turnover intention (β = −0.320, *t* = 3.634, *p* < 0.001); emotional engagement was negatively correlated to turnover intention (β = −0.403, *t* = 4.138, *p* < 0.001); and behavioral engagement was negatively correlated to turnover intention (β = −0.252, *t* = 2.982, *p* < 0.001).

In path analysis, the value of *R*^2^ indicates the explanatory ability of the model, explaining variation. Thus, the closer the value of *R*^2^ is to 1, the more powerful is the model’s explanatory ability. Awang et al. (2015) suggested that when *R*^2^ is larger than 0.67, the model would have good explanatory ability, around 0.33 indicates fair explanatory ability, and around 0.19 means poor explanatory ability.

Positive affect to cognitive engagement has good explanatory ability (*R*^2^ = 0.795); positive affect to emotional engagement has good explanatory ability (*R*^2^ = 0.762); positive affect to behavioral engagement has good explanatory ability (*R*^2^ = 0.714); and cognitive engagement, emotional engagement, and behavioral engagement to turnover intention also has good explanatory ability (*R*^2^ = 0.796).

According to Cohen (1988), the effect size Cohen’s *f*^2^ is defined as follows: Where *R*^2^ is the squared multiple correlation, *f^2^* ≥ 0.02, *f*^2^ ≥ 0.15, and *f*^2^ ≥ 0.35 represent small, medium, and large effect sizes, respectively. Although the above data can be used to determine statistical significance, if it is confirmed that it is practically significant, it can be judged by the verification of the effect quantity. Regarding the effect size in this study, the results showed that positive affect to cognitive engagement has a large effect size (*f*^2^ = 2.496); positive affect to emotional engagement has a large effect size (*f*^2^ = 3.201); positive affect to behavioral engagement has a large effect size (*f*^2^ = 3.878); and cognitive engagement, emotional engagement, and behavioral engagement to turnover intention also has a large effect size (*f*^2^ = 3.901).

#### Indirect Effect Analysis

When analyzing indirect effects, if the interval between the two values does not include zero, it means that the model has an indirect effect (Preacher and Hayes, 2008). The indirect effect of positive affect on turnover intention is between 0.180 and 0.379, and the indirect effect of WE on turnover intention is between 0.720 and 0.861. Each 95% confidence interval (CI) did not include zero, thus indicating that indirect effect existed in this research model (see Table 4). Results of this study provided initial evidence of the role of WE in the remote working context of intention to turnover regarding a positive affect trait. As hypothesized, a higher degree of positive affect was negatively related to turnover intention, thus supporting H7. That is, positive affect can negatively predict turnover intention mediated by the three types of work engagement.

**TABLE 4:** Indirect effect analysis.

Indirect effect	Positive affect		Remote WE	
	β	95% CI	β	95% CI
Turnover intention	−0.274**	[0.180, 0.379]	−0.802**	[0.720, 0.861]

**p < 0.01.

## Discussion

By examining how remote WEs play a mediating role in linking R&D professionals’ positive affect and their subsequent turnover intention during the COVID-19 lockdown, this study drew on the appraisal theories of emotion (Scherer et al., 2001) and ecological theory (Bronfenbrenner, 1995) to evaluate the effect of the environment on employees’ feelings while working remotely during the COVID-19 lockdown. The results of this study are discussed in more detail as follows.

From the perspective of positive psychology, positive affect plays an important role as a facilitator of engagement in a job (Laguna, 2019; Yan et al., 2019). For example, Perera et al. (2018) found that workers perceived that their behavioral, cognitive, and emotional engagements while performing a task were influenced by their positive or negative affect. Coo et al. (2021) identified the potential positive and negative perspectives related to congruence and incongruence between individuals and their teammates that affected their WE. Consistent with those studies, H1 was verified, indicating that participants’ positive affect was positively related to their remote cognitive engagement during the COVID-19 pandemic.

Additionally, Perera et al. (2018) showed that workers’ WE, including their cognitive engagement, will be promoted if they perceive their job as being significant. McLaughlin et al. (2019) pointed out that positive affect may lead to enhanced emotion regulation, while more positive emotions may have an impact on the extent of enhancing one’s capacity to notice positive emotions and experiences which occur in the workplace. In examining H2, the results showed that participants’ positive affect was positively related to their emotional engagement. Moreover, the higher their personal WE, and the less personal fatigue they feel, the more willing they are to quit (Ferreira et al., 2019). Supporting this study, the results showed that participants’ positive affect was positively related to their remote behavioral engagement, indicating that H3 was positively supported.

Work engagement is a multi-dimensional variable support associated with job demands. Consequently, employees have to manage multiple tasks at the same time in stressful working conditions, causing the turnover intention to withdraw from an organization (Park et al., 2019). That is, increasing employees’ cognitive engagement can effectively reduce their turnover intention (Lee M. C. C. et al., 2019). In examining H4, the results showed that participants’ cognitive engagement was negatively related to their turnover intention. Moreover, employees’ work emotion tends to make them more energized to perform well in their jobs, thus resulting in higher levels of emotional engagement at work and a lower level of turnover intention (Saks, 2021). In examining H5, the results showed that the participants’ emotional engagement was negatively related to their turnover intention. Additionally, Jain and Asawa (2019) suggested that engagement plays an important role in generating behavioral responses. In examining H6, the results showed that participants’ remote behavioral engagement was negatively related to their turnover intention, indicating that employees who have more behavioral engagement will show less turnover intention. Conclusively, positive cognitive, emotional, and behavioral engagement will reduce employees’ turnover intention during the COVID-19 pandemic. It can be noticed that workers can significantly improve their three types of engagement and reduce their willingness to leave with higher work pressure (Li et al., 2019), such as ICT R&D professionals working remotely.

The validated and reliable positive affect found an improvement role in viewing WE, and reducing intentions to leave amongst the commercial contract research organization samples (Heyns, 2021). When perceiving organizational support as a positive factor, WE can mediate the relationship between a high level of positive affect and organizational commitment (Teo et al., 2020). However, employees felt that engagement of all specialties with the work process had decreased when working from home. Those with lower levels of positive affect tend to change their jobs during the period of working from home (Mohamedbhai et al., 2021). In examining H7, the results showed that the R&D professionals’ positive affect was negatively related to their turnover intention, mediated by their remote WE during COVID-19.

## Conclusion

With the rapid spread of COVID-19, how to keep R&D professionals in organizations has become a common concern. When proposing the expanded person–job fit model, positive affect could lead to the sense of job-fit and work-based engagement. The results of this study reveal the correlation between positive affect, WE, and turnover intention, in which one can understand the significance of the empowerment of positive affect for R&D professionals to decrease their turnover intention. That is, the positive affect of R&D personnel had significant predictive power on the three types of WE, and all three types of WE of R&D personnel had negatively predictive power for turnover intention in the ICT industry.

### Implications

The results can be applied to enhance workers’ engagement so as to reduce their intention to change jobs by selecting R&D workers with a high level of positive affect. The results can also help employers who want to understand what factors will help their workers reduce their turnover intention by increasing their positive affect when facing the crisis of the COVID-19 pandemic.

Gottschalck et al. (2020) highlighted that if organizations can consider individual differences in the patterns of turnover intentions for different types of workers, they can design incentive strategies that are needed and closely aligned with those workers’ specific needs. Supporting their study, the results of the present study suggest that ICT human resource managers can use incentives to strengthen remote WE that is necessary to keep R&D professionals working remotely.

### Contribution

During the outbreak of the COVID-19 pandemic, along with lockdowns and working from home, some employees’ work attitude toward engaging in their work has changed and has consequently influenced the degree of their turnover intention. This phenomenon is also potentially influencing the practice of human resources, in particular, the cost of personnel training and management. Drawing on job demand-resource theory, this study aimed to elaborate the correlation between the psychologically positive affect, work engagement, and turnover intention of R&D professionals in the ICT Industry. Through structural equation modeling, we found that positive affect can positively predict three types of employees’ remote work engagement and negatively predict their turnover intentions. There are two principal contributions of this study. First, theoretically, the study introduced the job demand-resource theory to explore the psychological traits affecting psychological adjustment in working from home during COVID-19, in particular, for the long-term telecommuting R&D IT staff. Second, the results of this study can be applied to human resource management to reduce the intention to leave of R&D staff in the IT industry and to decrease HR costs by selecting R&D professionals with positive emotion, thus increasing their work engagement.

### Limitations and Future Study

Future research could be conducted as follows. The workplace and work content of employees in the ICT industry, based on employees’ job demands-resources, are different from each job. Thus, besides R&D professionals, future studies related to factors affecting turnover intention may consider a cross-sectional study of the ICT industry to understand the predicting power of the antecedents on turnover intention. Moreover, the advancement of technology is undergoing extremely rapid change in the ICT industry, meaning that the work content of R&D professionals is constantly changing. To understand how this change affects employees’ turnover intention over time, a longitudinal study may be worthwhile.

In this study, the participants were R&D workers who were mainly less than 40 years old and who had a baccalaureate degree. They were working in the area of software R&D. Dalton et al. (1999) suggested that age, work experience, and geographic preferences are differential factors related to employees’ turnover intention. However, differentiation analysis regarding turnover intention was not performed; thus, future studies can explore the differential power of age, gender, work experience, and so on. Integrated Circuit engineers can also be selected as the research sample.

Moreover, turnover research may benefit from studying the role of emotional self-efficacy beliefs (i.e., people’s perceived capability and confidence to successfully manage their emotions; Caprara et al., 2013) as they have been already shown consistent evidence in negatively predicting turnover intentions in the workplace (Cepale et al., 2021). Accordingly, it may be worthwhile, to test emotional self-efficacy beliefs as a further mechanism able to explain turnover intentions among ICT workers.

Instead of focusing on studying what factors affect turnover intention, Hom et al. (2017) studied the psychology of staying (rather than leaving) and attitudinal trajectories in predicting turnover, and suggested some human resource management strategies to promote staying. This alternative perspective of exploring why workers intend to stay can be taken and compared with those studies on intention to leave in future research.

## Data Availability Statement

The original contributions presented in the study are included in the article/supplementary material, further inquiries can be directed to the corresponding author.

## Author Contributions

J-CH and SP: concept and design. J-CH and YC: acquisition of data. J-CH, SP, and YC: drafting of the manuscript. J-CH and YC: critical revision of the manuscript. YC and SP: statistical analysis. All authors contributed to the article and approved the submitted version.

## Conflict of Interest

The authors declare that the research was conducted in the absence of any commercial or financial relationships that could be construed as a potential conflict of interest.

## Publisher’s Note

All claims expressed in this article are solely those of the authors and do not necessarily represent those of their affiliated organizations, or those of the publisher, the editors and the reviewers. Any product that may be evaluated in this article, or claim that may be made by its manufacturer, is not guaranteed or endorsed by the publisher.
